# Human Neutrophil Elastase: Characterization of Intra- vs. Extracellular Inhibition

**DOI:** 10.3390/ijms25147917

**Published:** 2024-07-19

**Authors:** Denise Birk, Erika Siepmann, Stefan Simon, Christian P. Sommerhoff

**Affiliations:** 1 Institute of Laboratory Medicine, University Hospital, LMU Munich, 80336 Munich, Germany; 2 Department of Neurology, LMU University Hospital, LMU Munich, 81377 Munich, Germany; 3 Institute of Medical Education, University Hospital, LMU Munich, 80336 Munich, Germany

**Keywords:** neutrophils, neutrophil elastase, elastase inhibitors

## Abstract

Neutrophil elastase (HNE), like other members of the so-called GASPIDs (Granule-Associated Serine Peptidases of Immune Defense), is activated during protein biosynthesis in myeloid precursors and stored enzymatically active in cytoplasmic granules of resting neutrophils until secreted at sites of host defense and inflammation. Inhibitors thus could bind to the fully formed active site of the protease intracellularly in immature progenitors, in circulating neutrophils, or to HNE secreted into the extracellular space. Here, we have compared the ability of a panel of diverse inhibitors to inhibit HNE in the U937 progenitor cell line, in human blood-derived neutrophils, and in solution. Most synthetic inhibitors and, surprisingly, even a small naturally occurring proteinaceous inhibitor inhibit HNE intracellularly, but the extent and dynamics differ markedly from classical enzyme kinetics describing extracellular inhibition. Intracellular inhibition of HNE potentially affects neutrophil functions and has side effects, but it avoids competition of inhibitors with extracellular substrates that limit its efficacy. As both intra- and extracellular inhibition have advantages and disadvantages, the quantification of intracellular inhibition, in addition to classical enzyme kinetics, will aid the design of novel, clinically applicable HNE inhibitors with targeted sites of action.

## 1. Introduction

Neutrophils (PMN) play a critical role in innate immunity, providing the first line of defense against bacterial and viral infections but also contribute to initiating and maintaining acute and chronic inflammatory disorders. Key mediators of neutrophil-driven inflammation include the so-called GASPIDs (Granule Associated Serine Peptidases of Immune Defense), a group of serine proteases that in neutrophils comprise human neutrophil elastase (HNE), cathepsin G, proteinase 3 and the more recently described neutrophil serine protease 4 (NSP4, PRSS57) [1,2,3,4]. Unlike most other proteases, which are expressed or activated from dormant zymogens on demand, GASPIDs are expressed and immediately activated by cathepsin C/dipeptidyl peptidase I during neutrophil maturation and subsequently stored fully enzymatically active in azurophilic granules [5,6]. Neutrophil GASPIDs thus contribute to neutrophil (patho)physiology not only extracellularly after being released from activated neutrophils but also by acting intracellularly, participating, e.g., in the killing of ingested pathogens [2,3,7,8].

Among the GASPIDs, HNE has consistently been considered a pivotal target in drug development addressing neutrophil-mediated disorders. Due to its ability to cleave a wide variety of structural and regulatory proteins, including cytokines and cell surface receptors, an imbalance between released HNE and its endogenous inhibitors (e.g., plasma-derived α_1_ proteinase inhibitor (α1-PI) and α_2_ macroglobulin, tissue-derived antileukoproteinase/secretory leukocyte protease inhibitor and elafin) can cause tissue injury and inflammation. Initially implied in emphysema and chronic obstructive pulmonary disease (COPD), HNE’s involvement has subsequently expanded to acute and chronic disorders, including, e.g., acute respiratory distress syndrome (ARDS), sepsis, rheumatoid arthritis, inflammatory bowel disease, cancer, and recently even COVID-19. Since the first formulation of the protease–antiprotease hypothesis of emphysema pathogenesis in the 1960s [9], a large variety of synthetic and proteinaceous HNE inhibitors have been investigated for their therapeutic efficacy [10,11,12,13,14]. Disappointingly, however, besides purified human α1-PI for treating α_1_ antitrypsin deficiency [15] only two inhibitors have received clinical approval for very limited applications, namely the synthetic inhibitors alvelestat/MPH-966/AZD9668 (designated orphan drug status for treating α_1_ antitrypsin deficiency [16]) and sivelestat/ONO5046 (approved in some Asian states for treating ARDS, systemic inflammatory response syndrome (SIRS), and COVID-19 [17,18,19]). Both compounds, however, have not been approved in other countries or for other indications due to limited efficacy [20,21,22].

In contrast to the endogenous proteinaceous antiproteases that inhibit HNE extracellularly, synthetic inhibitors, due to their lower molecular mass, have the additional potential to penetrate PMN [23] and inhibit HNE within azurophilic granules. They may thus attenuate intracellular functions of HNE and lead to the secretion of a transiently or irreversibly inhibited HNE by activated PMN. Here, we have systematically investigated the intracellular inhibition of HNE using both a human myeloid progenitor cell line and human mature peripheral blood-derived PMN. The results show that the extent and dynamics of intracellular inhibition differ substantially from those obtained by classical steady-state and pre-steady-state inhibition kinetics describing extracellular inhibition. Surprisingly, both alvelestat and sivelestat, i.e., the abovementioned two compounds whose efficacy is disputed, form complexes with HNE that dissociate rapidly and are the only synthetic inhibitors that do not cause detectable intracellular inhibition in our cell culture model. Thus, by combining enzyme kinetics with these cell culture models, both extracellular and intracellular inhibition can be assessed to aid the design and development of more effective GASPID inhibitors for the treatment of neutrophil-mediated disorders that have been disappointing over the past six decades.

## 2. Results

### 2.1. Quantification of Elastase-like Activity in Mature Human Neutrophils and Myeloid Precursor Cell Lines

**Figure 1:**
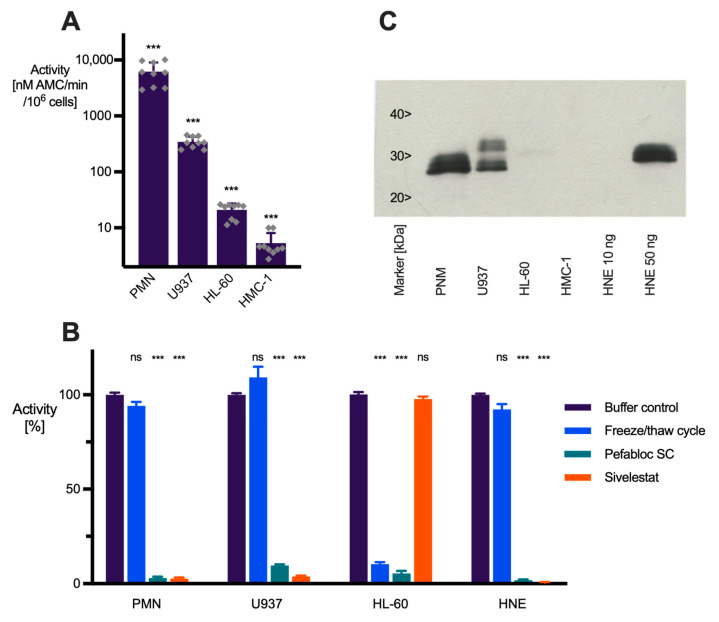
Characterization of HNE-like activity in mature human neutrophils and human myeloid precursor cell lines. (**A**) HNE-like activity was measured in cell lysates of isolated peripheral PMN and the cell lines U937, HL-60, and HMC-1 using the fluorogenic substrate MeOSuc-AAPV-AMC. HNE-like activity is expressed in nM AMC/min/10^6^ cells based on an AMC standard curve. Both mean ± SEM and individual values from 3 individual experiments with three technical replicates each are shown; ***, *p* ≤ 0.0001 to all other groups. (**B**) HNE-like activity in cell lysates is measured compared to isolated HNE (0.5 nM) after incubation (1 h, 37 °C) with buffer, the serine protease inhibitor Pefabloc SC (1 mM), or the elastase-specific inhibitor sivelestat (4 µM), or after one freeze/thawing-cycle. Data are mean ± SEM, *n* ≥ 3; ***, *p* ≤ 0.0001; ns, no inhibition. (**C**) Detection of HNE by western blot in lysates of human PMN (100,000 cells/lane), U937, HL 60, and HMC-1 cells (500,000 cells/lane each). 10 and 50 ng isolated HNE were used as positive controls. The blot is representative of 3 independent experiments.

To identify a cell line suitable to study the effects of HNE protease inhibitors in proliferating immature neutrophil progenitors, we quantified the elastase-like activity in HL-60, the most commonly used cell line in neutrophil research [24], and in the myeloid cell line U937, which can differentiate into neutrophils [25] and expresses HNE [5,26,27]. As a model of myeloid precursors, proliferating cells were used without inducing any further differentiation, e.g., by retinoic acid or DMSO. Elastase-like activity was measured in cell lysates using the fluorogenic substrate MeOSuc-AAPV-AMC [28]. Isolated human neutrophils and the human mast cell line HMC 1 [29] were used as the gold standard and negative control, respectively. Elastase-like enzymatic activity is detectable in PMN-, U937-, and HL-60-lysates but only at the detection limit in HMC-1 lysates (Figure 1A). Using isolated HNE as a standard, the elastase-like activity in PMN was calculated to correspond to 1.0 ± 0.5 µg HNE per 10^6^ PMN, which is in accordance with literature data [30,31,32]. For U937 cells the calculated HNE content is about 20-fold lower (0.06 ± 0.01 µg per 10^6^ cells). Importantly, the variability of activity measurements is smaller in cultured U937 cells than PMN, which may be in part due to the 10 to 20 fold higher number of cells used per assay. Moreover, elastase-like activity in the U937 cell line can be determined repeatedly and reliably throughout culture in microtiter plates for up to 26 h.

### 2.2. Elastase-like Activity in PMN and U937 Cells Is Due to the Presence of HNE

Next, we investigated whether the elastase-like activity is due to the presence of HNE or other proteases with similar enzymatic activity. Therefore, elastase-like activity was measured in cell lysates after incubation with the general serine protease inhibitor Pefabloc SC or the HNE-specific inhibitor sivelestat and compared to the inhibition of isolated HNE (0.5 nM) by these compounds. Pefabloc SC (1 mM) inhibits the activity of isolated HNE as well as those in all lysates (inhibition 98 ± 0.6%, 97 ± 0.6%, 91 ± 0.6%, and 95 ± 1% for HNE, PMN, U937, and HL-60 cells, respectively; Figure 1B), verifying the presence of a serine protease. The HNE-specific inhibitor sivelestat (4 µM), however, inhibits the activity of isolated HNE and that present in PMN- and U937-lysates (99 ± 0.3%, 97 ± 0.6%, and 96 ± 0.3%, respectively), but has little effect on the activity in HL-60 lysates (‘inhibition’ 2 ± 1.2%). Moreover, isolated HNE and the elastase-like activity in PMN- and U937-lysates share other properties, such as stability over time and freeze/thawing cycles, whereas activity in HL-60 lysates rapidly diminishes over time and lacks cryostability (Figure 1B). Finally, western blot verified the presence of HNE in PMN- and U937-lysates, whereas little if any immunoreactivity is detectable in HL-60 and HMC-1 cells used as a control (Figure 1C). Taken together, these results suggest that the elastase-like activity in PMN- and U937-lysates is due to the presence of HNE, whereas HL-60 cells contain a distinct serine proteinase that is capable of cleaving the ‘elastase substrate’ MeOSuc-AAPV-AMC.

### 2.3. Isolated HNE and HNE in U937 Lysates Have Identical Inhibition Profiles

To further characterize HNE in U937 lysates compared to isolated HNE, we utilized a panel of 13 proteinase inhibitors, which includes inhibitors ranging from small synthetic compounds such as the generic serine proteinase inhibitor Pefabloc SC and the selective, clinically approved HNE inhibitors alvelestat/MPH-966/AZD9668 and sivelestat/ONO5046 to much larger naturally occurring proteinaceous inhibitors such as eglin C and α1-PI. The inhibitors’ affinity to isolated HNE and HNE in U937 lysates was assessed by determining the equilibrium dissociation constant *K*_i_ of the complex and the IC_50_ value at 60 min for reversible and irreversible inhibitors, respectively (Table 1). The affinity of the individual compounds to isolated HNE differs largely, with *K*_i_-values ranging from 1.3 ± 0.2 × 10^−11^ M (eglin C) to 3.9 ± 0.4 × 10^−7^ M (BPTI) and IC_50(60min)_-values ranging from 4.6 ± 1.8 × 10^−10^ M (α1-PI) to 1.3 ± 0.1 × 10^−4^ M (Pefabloc SC). Although the individual affinities to HNE differ 10^7^-fold, the inhibitors have a comparable affinity to both isolated HNE and HNE in U937 lysates (average difference between corresponding values 18% for all inhibitory compounds except eglin C, see below). These virtually identical inhibition profiles verify that the elastase-like activity in U937-lysates is due to the presence of HNE. Furthermore, the results also show that other components present in U937-lysates have little influence on the affinity of most inhibitors towards HNE and on the determination of *K*_i_ and IC_50_ values. One notable exception is eglin C, which inhibits HNE in cell lysates 10-fold less effectively than isolated HNE (*K*_i_ 1.9 ± 0.8 × 10^−10^ M vs. 1.3 ± 0.2 × 10^−11^); most likely, the effective concentration of eglin C is diminished by binding to other lysate components, e.g., cathepsin G and proteinase 3, which is particularly relevant for this high-affinity compound that is used in minute concentrations. As expected, the negative controls, benzamidine, and cathepsin G inhibitor, showed no inhibition of HNE.

### 2.4. Effect of Inhibitors on HNE in the Immature Precursor Cell Line U937

**Figure 2:**
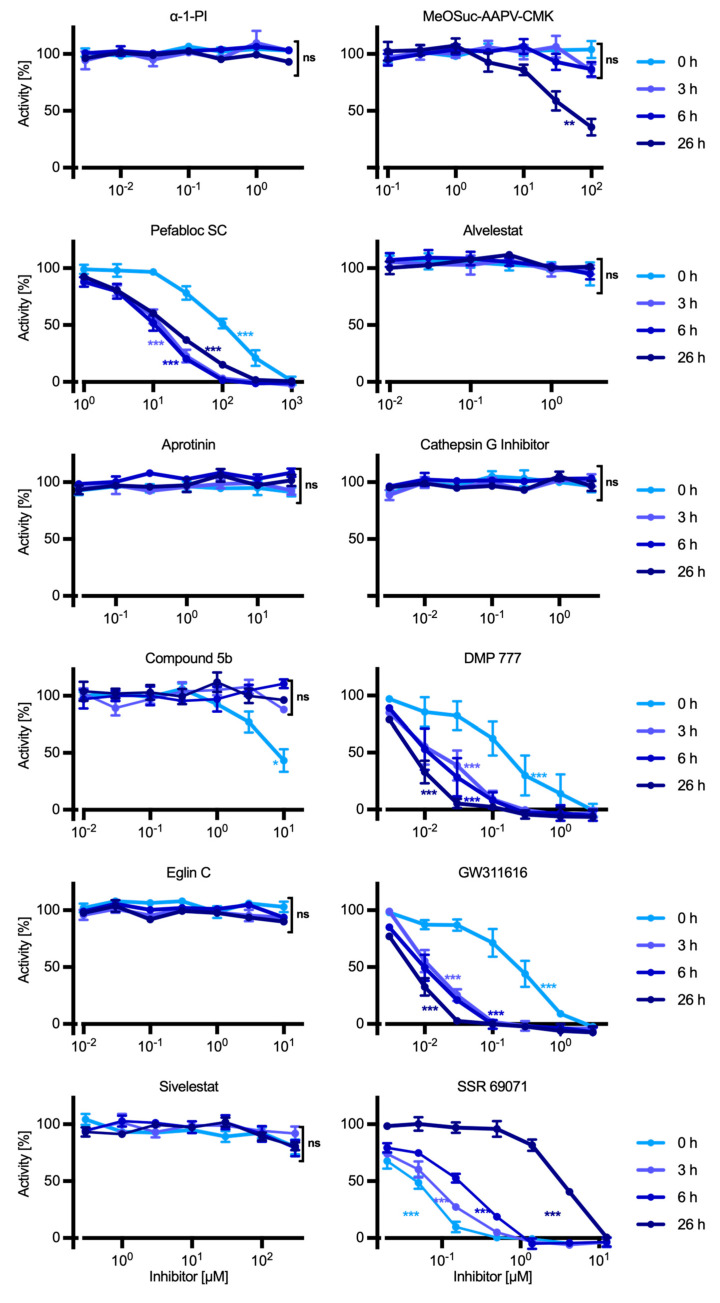
Effect of inhibitors on HNE in U937 cells as a model of immature neutrophils. Cells were cultured for 0–26 h with increasing concentrations of the compounds or solvent controls. Subsequently, cells were washed and lysed, and residual HNE activity was measured using the HNE substrate MeOSuc-AAPV-AMC. Residual activity is expressed as a percentage of matched solvent controls. Data are mean ± SEM, *n* ≥ 3; ***, *p* ≤ 0.0001; **, *p* ≤ 0.001; *, *p* ≤ 0.05; ns, not significant.

To evaluate the effects of protease inhibitors on intracellular HNE in immature PMN precursors, U937 cells were plated in 96-well plates and cultured for 26 h. Increasing concentrations of inhibitors and controls were added at different points in time before harvesting and quantification of residual HNE activity in cell lysates. Among all inhibitors studied, only Pefabloc SC, i.e., the compound with the smallest molecular mass (240 Da), shows comparable inhibition using intact U937 cells and isolated HNE, with an IC_50_ of ~10^−4^ M and nearly complete inhibition at ~10^−3^ M in both systems (Figure 2). Inhibition in the cell culture model increases during the first 6 h (IC_50_ ~10^−4^ M and ~10^−5^ M at 0 and 6 h, respectively), consistent with an ongoing reaction of the irreversible inhibitor with HNE. Subsequently, inhibition decreases slightly at 26 h, likely reflecting the synthesis of new HNE by U937 cells that is not any more inhibited by Pefabloc SC due to the compounds’s limited stability [33].

Like Pefabloc SC, the other irreversible synthetic compound studied, MeOSuc-AAPV-CMK, as well as the reversible compounds DMP 777 and GW311616, inhibit HNE in U937 cells increasingly over the 26 h observation period and thus surprisingly slowly (see below). Compound 5b and SSR 69071, however, are most effective immediately after addition and then lose effect over time, suggesting that these compounds are rapidly inactivated in the cell culture model. All compounds inhibit HNE in the cell culture model less effectively than isolated HNE, with IC_50_-values 10- to 10,000-fold higher than the IC_50_- and *K*_i_-values measured by enzyme kinetics (Table 1).

As expected, the proteinaceous inhibitors α1-PI, eglin C, and BPTI do not inhibit HNE in the U937 model, in agreement with the limited ability of macromolecular compounds to enter cells [23]. Surprisingly, however, also alvelestat and sivelestat have no detectable effect on the activity of HNE in the U937 cell culture model even at concentrations more than 1000-fold above *K*_i_. These results suggest that these compounds either cannot enter cells although the molecular mass of these synthetic compounds (546 and 434 Da) is in the range of effective compounds, are very unstable and rapidly lose activity, or only temporarily inhibit HNE due to limited stability of the HNE/inhibitor complex.

### 2.5. Effect of Inhibitors on HNE in Mature Human Neutrophils

To compare the inhibition of HNE in the immature U937 cell line and mature PMN, cells were isolated from the venous blood of healthy donors and incubated with selected inhibitors. Due to the limited lifespan of mature PMN that rapidly undergo apoptosis after isolation from blood [34], the incubation time was shortened from 3 h to 1 h. Also, cell numbers were adjusted to compensate for the higher amounts of HNE in differentiated PMN compared to U937 cells (see above). Cell lysis and quantification of HNE were performed as completed for U937 cells.

**Figure 3:**
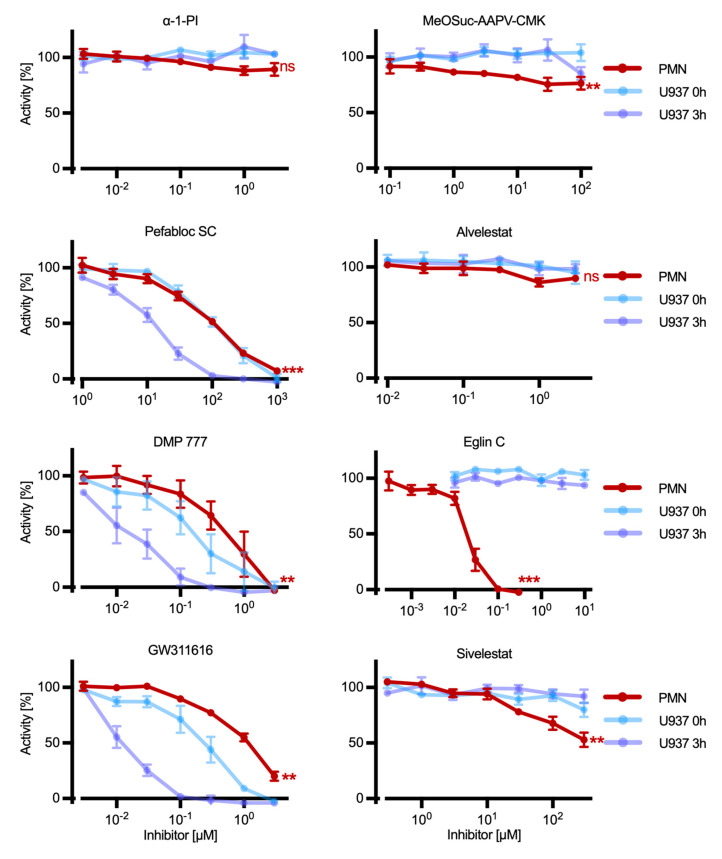
Effect of inhibitors on HNE in mature PMN. Freshly isolated PMN were incubated for 1 h with increasing concentrations of the inhibitors or solvent controls. Subsequently, cells were washed and lysed, and residual HNE activity was measured using the HNE substrate MeOSuc-AAPV-AMC. Residual activity is expressed as a percentage of matched solvent controls. Data are mean ± SEM, *n* ≥ 3; ***, *p* ≤ 0.0001; **, *p* ≤ 0.001; ns, not significant. For comparisons, the effects of the inhibitors on U937 cells (0–3 h, see Figure 2) are shown in different shades of blue.

For most of the inhibitors, the effects on HNE observed using mature PMN and immature U937 cells are very similar (Figure 3). In particular, the concentration-dependent HNE inhibition by Pefabloc SC is virtually identical in PMN and U937 cells (IC_50_ each ~10^−4^ M). Most compounds, particularly sivelestat, are somewhat more effective in PMN than U937 cells, whereas GW311616 and DMP 777 appear somewhat less potent. A notable exception is eglin C, which inhibits HNE in PMN with an IC_50_ of ~20 nM, while it shows little effect on HNE in U937 cells even at 10 µM after 26 h. Thus, it appears that this naturally occurring compound can access HNE in granules of mature PMN despite its relatively high mass (8091 Da).

### 2.6. Formation and Stability of the HNE/Inhibitor Complex

As the dynamics of the HNE inhibition in the U937 and PMN cell models appeared slow, we next assessed the kinetics of the formation and dissociation of the complex of the inhibitors with isolated HNE, which describes extracellular inhibition. Therefore, the association rates *k*_on_ of all compounds were measured using pre-steady-state kinetics. Subsequently, the dissociation rates *k*_off_ for reversible inhibitors were calculated as *k*_off_ = *K*_i_ × *k*_on_ using the *K*_i_ determined under identical conditions (see above and Table 1); irreversible inhibitors form a stable complex so that *k*_off_ is close to zero. Finally, the halftime of the HNE/inhibitor complex formation and dissociation were calculated as t_1/2,on_ = 0.693/(*k*_ass_ × I_t_ + *k*_off_) and t_1/2,off_ = 0.693/*k*_off_, respectively.

Measured association rates of the 10 compounds studied (Table 2) vary more than 10^6^-fold from 1.0 ± 0.1 M^−1^ s^−1^ (Pefabloc SC) to 2.9 ± 0.09 × 10^6^ M^−1^ s^−1^ (eglin C); for alvelestat, association (7 × 10^6^ M^−1^ s^−1^ according to [35]) is even faster and too fast to be determined precisely using our experimental setup. With these association rates, all compounds except Pefabloc SC should inhibit HNE by ≥50% in less than 10 s when applied at the highest concentration in the U937 cell model (Table 2). Thus, the much slower onset of inhibition observed in the cell culture model is obviously due to factors such as diffusion barriers to access HNE within granules.

Calculated dissociation rates of the HNE complex with reversible inhibitors differ by three orders of magnitude between 5.7 × 10^−2^ s^−1^ for alvelestat [35] and 3.8 × 10^−5^ s^−1^ for eglin C. Three compounds, i.e., alvelestat, sivelestat, and compound 5b, form complexes with HNE that have half-lives of only seconds to a few minutes, whereas the complexes with other inhibitors are stable for a prolonged time. Thus, even if alvelestat, sivelestat, and compound 5b access HNE in cells, the complexes are unstable and will dissociate during cell harvesting and lysis—and, similarly, after degranulation in vivo.

### 2.7. Inhibitor Stability

As described above, Pefabloc SC, compound 5b, and SSR 69071 lose effectiveness over time in the U937 cell culture model, suggesting that these compounds are unstable or metabolized. Therefore, we determined the stability of all compounds by comparing their ability to inhibit HNE before and after incubation in a cell culture medium. Measurements were performed using a serum-free medium because a complete medium interfered with activity measurements. After 26 h of incubation, the inhibitory activity of Pefabloc SC and compound 5b decreased by approximately 50 and 90%, which explains that these compounds are most effective immediately after addition to the cell culture model and that HNE inhibition decreases slightly over time (see Supplementary Figure S1). SSR 69071 and all other inhibitors appeared stable under these conditions; we cannot exclude, however, that these compounds are inactivated by serum components or are metabolized by U937 cells.

### 2.8. Inhibitor Toxicity in U937 Cells and PMN

To check for toxic effects of the inhibitors, the proliferation of U937 cells and the LDH release from both U937 cells and PMN into the culture medium was monitored. At the highest concentrations used, Pefabloc SC (1000 and 300 µM) and SSR 69071 (12.5 µM) are slightly toxic in U937 cells (12 ± 2% LDH release each after 26 h; Supplementary Figure S2A). Equivocal, Pefabloc SC in concentrations ≥300 µM reduced cell proliferation by 50 ± 3%. Lower concentrations of these compounds and all other inhibitors, however, had no detectable effects on LDH release and U937 proliferation, suggesting no overt toxicity. During the much shorter exposure of PMN (1 h), no significant LDH release was detectable (Supplementary Figure S2B).

## 3. Discussion

Unlike other proteases that are stored as zymogens, neutrophil elastase (HNE) is expressed in myeloid progenitor cells and immediately processed to its active form in the trans-Golgi before being targeted to azurophil granules. HNE, therefore, is fully enzymatically active when stored in granules of myeloid progenitors, in resting and circulating neutrophils, and when secreted by stimulated neutrophils into the extracellular space. In the current study we have shown that synthetic inhibitors and even the small leech-derived inhibitor eglin C have the potential to interact with the active site of HNE and to inhibit the protease throughout all these stages. The extent and kinetics of intracellular inhibition, however, are quite different from and not predicted by classical enzyme kinetics that describe inhibition in solution.

In search of a readily available alternative to human bone marrow, we have screened several hematopoietic progenitor cell lines for the presence of HNE. Cultured U937 and HL-60 cells both were found to contain elastase-like activity, although at levels approximately 20- and 300-fold lower, respectively, than mature blood-derived PMN, consistent with previous studies [26,36]. Subsequent western blot analysis and inhibitor profiling confirmed that the elastase-like protease in U937 cells and isolated PMN is HNE. In contrast, the activity in HL-60 lysate resists inactivation by HNE inhibitors; it is thus due to a different serine elastase, potentially one of the mutant forms of HNE detected in severe congenital neutropenia that have altered affinity to α1-PI [37]. Although HL-60 is one of the commonly used cell lines in neutrophil research, it should thus not be used for studies related to HNE and probably other GASPIDs. On the contrary, the U937 cell line proved to be a reliable cell culture model in our hands, allowing repeated measurements of HNE activity with low variability even when cultured in microtiter plates for up to 26 h.

To compare the inhibition of HNE in cells and in solution, we selected a panel of 13 diverse inhibitors and controls, including synthetic and proteinaceous compounds with molecular masses ranging from 240 Da to 52 kDa and with reversible and irreversible inhibition mechanisms. The affinity towards HNE and the association kinetics obtained with isolated HNE in a physiological buffer supported the diversity of the panel: The compounds cover a wide range of affinities (*K*_i_ and IC_50_ values 13–130 μM), association rates (1 M^−1^s^−1^–7 × 10^6^ M^−1^s^−1^) and dissociation rates (0–0.057 s^−1^). The kinetic parameters obtained for most inhibitors are in good agreement with literature values, although some differences were noted, particularly for MeOSuc-AAPV-CMK, DMP 777, and SSR 69071, likely reflecting assay conditions and the purity of the commercial compounds. Subsequently, the kinetic parameters *K*_i_ and IC_50_ were determined a second time using the HNE activity in U937 cell lysates. The almost identical values thus obtained show that cellular components in (diluted) PMN lysates have little on measurements of HNE activity, inhibition kinetics and reproducibility. A major exception is eglin C, which is ~10-fold less potent in PMN lysates, probably because eglin C can also bind to many other lysate components, including cathepsin G and proteinase 3 [38], thus diminishing the effective inhibitor concentration.

Exposure of cultured U937 cells and freshly isolated PMN revealed that most of the synthetic compounds penetrate cells and inhibit HNE intracellularly, as expected from some previous studies with single compounds (i.e., GW311616, DMP 777, sivelestat) [39,40]. Only the compound with the lowest molar mass, Pefabloc SC (240 Da), inhibited intracellular and isolated HNE with similar affinity and time course. Most compounds (i.e., MeOSuc-AAPV-CMK, DMP777, GW311616, SSR 69071, and compound **5b**, 261–565 Da) are 100- to 10,000-fold less effective in cells. Thus, besides the inhibitor’s affinity, other factors such as the compartmentalization of HNE within the acidic and proteoglycan-rich milieu of azurophilic granules, diffusion barriers, and, in part, the limited stability of the inhibitors have a major influence on their efficacy in inhibiting HNE intracellularly. Two synthetic compounds, sivelestat and alvelestat, had no measurable effect on HNE activity in U937 lysates and a negligible effect on HNE in PMN lysates, likely due to a limitation of the current experimental approach (see below).

The proteinaceous inhibitors α1-PI and BPTI have no effect on intracellular HNE, consistent with the limited ability of macromolecules to enter cells [23]. Surprisingly, however, eglin C, a small leech-derived protein (70 amino acids, 8091 Da) that attracted interest due to its anti-inflammatory properties [38], inhibits intracellular HNE but only in mature neutrophils. Eglin C was previously shown to bind to the surface of neutrophils by a mechanism not involving HNE or other proteases, and some data even suggested internalization [41]. Interestingly, the surface binding affinity reported by Braun and Schnebli (K_d_ 0.2 µM) is in the same range as the IC_50_ for the inhibition of intracellular HNE (0.02 µM). It is tempting to speculate that eglin C has evolved the ability to inhibit intracellular HNE as a means to control inflammation and itching at the site of the bite, allowing the leech to feed on its host undetected. How this small protein penetrates differentiated PMN selectively and whether this mechanism is applicable to other compounds is currently under investigation.

Overall, our approach of assessing the extent and dynamics of intracellular HNE inhibition by measuring the residual activity in lysates of treated and untreated U937 and blood-derived human neutrophils appears appropriate for most of the compounds studied. The lack of effect of α1-PI in both cell models and the differences between intracellular and extracellular inhibition observed for most compounds confirm that the carryover of test compounds from the culture medium to the lysate is negligible. The results are consistent with a single pool of intracellular HNE in both cell models; potentially, the model can be extended to activated neutrophils to assess HNE exposed on the cell surface or in neutrophil extracellular traps. Importantly, however, the current approach is only applicable to compounds that form a relatively stable complex with HNE (*k*_off_ < 10^−3^ s^−1^, t_1/2,off_ > 10 min); the complexes with other inhibitors (e.g., alvelestat, sivelestat, and compound 5b in the current study) dissociate during lysate preparation and enzyme activity measurements, leading to an underestimation of intracellular inhibition. This limitation can be circumvented by reducing the lysate preparation time and the dilution factor; with a cell volume of ~300 µm^3^ [42], 10 million neutrophils comprise a total volume of ~3 µL, which even undiluted is sufficient for measurements in 1536-well plates or a nanophotometer (e.g., Thermo Scientific NanoDrop™). Even more attractive are in situ measurements of HNE activity before and during inhibitor exposure of living cells, e.g., by flow cytometry. Kasperkiewicz et al. have developed activity-based probes for elegantly imaging GASPIDs in live neutrophils [32,43]. However, these probes essentially are irreversible inhibitors and compete with reversible inhibitors, again resulting in an underestimation of inhibition as described above for dilution. In contrast, cell-permeable HNE substrates, when used at concentrations well below the K_m_, would not affect the protease/inhibitor equilibrium. For this purpose, the rhodamine-based AAPV Elastase CellProbe^TM^ reagent was propagated by Beckman Coulter but has been discontinued. We are currently screening whether this reagent, related rhodamine substrates such as (Z-AAAA)^2^Rh_110,_ or other substrates can be used to load neutrophils.

Should drug design target HNE extracellularly or in neutrophils? Our results can perhaps be interpreted that this depends on whether a compound is intended to prevent or treat inflammation. Compounds targeting extracellular HNE mimic physiological antiproteases such as α1-PI and are likely to have little mechanism-based side effects. Unfortunately, their effectiveness in inhibiting HNE released by activated neutrophils at the site of inflammation is largely dependent on a variety of factors, including their local concentration, the association rate, and the milieu; in particular, a protein- and substrate-rich environment such as inflammation can render synthetic (usually competitive) inhibitors ineffective. Compared to purely extracellular-acting inhibitors, compounds that inhibit HNE intracellularly in neutrophils could have more on-target side effects because they attenuate intracellular functions of HNE [8]. However, the absence of active HNE in PMN is tolerated, as seen in people with the congenital cathepsin C deficiencies Haim-Munck and Papillon-Lefèvre syndrome, in the clinical use of the HNE inhibitors alvelestat and sivelestat [20,21], and in clinical trials of novel cathepsin C inhibitors [44,45,46]. Compounds that inhibit intracellular HNE could act slowly over a prolonged time during PMN maturation or in circulation, making the compound’s concentration and the milieu at the site of inflammation irrelevant. Such ‘preloading’ of PMN with (quasi) irreversible inhibitors that could even be dosed intermittently results in the release of an ‘enzymatically dead’ HNE species at the site of inflammation and will thus be highly effective. ‘Preloading’ of PMN with compounds whose complex with HNE dissociates rapidly (high *k*_off_), however, will be much less effective as the inhibitor/HNE complex dissociates at the site of inflammation; thus, regenerating active HNE and an inhibitor that shares all the limitations of extracellular acting compounds discussed above.

In summary, based on our results and the considerations discussed above, compounds whose complex with HNE dissociates rapidly (high *k*_off_) will be less effective for treating inflammation, a shortcoming that may be the main reason for the limited clinical efficacy of sivelestat and alvelestat. Compounds with properties like eglin C, i.e., (quasi) irreversible inhibitors that penetrate neutrophils to inhibit HNE in azurophilic granules, are likely to be much more effective in treating inflammation in a clinical setting. Our approach, combining classical inhibition kinetics with cell models of immature and mature neutrophils to assess both extracellular and intracellular inhibition, may foster the development of such effective and clinically applicable HNE inhibitors.

## 4. Materials and Methods

### 4.1. Reagents

The HNE inhibitors alvelestat/MPH-966/AZD9668 (HY-15651), DMP 777 (HY-75957) and GW311616 (HY-15891) were obtained from Hycultec (Beutelsbach, Germany), sivelestat/ONO5046 (127373-66-4) from Sigma-Aldrich (Taufkirchen, Germany), compound 5b (Cay14922-10) from Biomol (Hamburg, Germany), SSR 69071 (2506/10) from R&D Systems (Wiesbaden, Germany) and MeOSuc-AAPV-CMK (324745) from Merck (Darmstadt, Germany). Eglin C was a gift from Ciba-Geigy (Basel, Switzerland). α_1_-protease inhibitor (ab91136) and cathepsin G inhibitor (ab142181) were from Abcam (Cambridge, UK), benzamidine (B-6506) from Sigma-Aldrich (Taufkirchen, Germany), aprotinin (bovine pancreatic protease inhibitor, BPTI, Trasylol^®^) from Bayer (Leverkusen, Germany) and Pefabloc SC/AEBSF (124839) from Merck (Darmstadt, Germany). Human neutrophil elastase (HNE) (324681) was purchased from Merck (Darmstadt, Germany), and the substrate MeOSuc-AAPV-AMC (4005227) from Bachem (Bubendorf, Switzerland). 7-Amino-4-methylcoumarin (164545) was purchased from Sigma-Aldrich (Taufkirchen, Germany). Cell culture media for U937 cells (RPMI 1640 with L-Glutamine and Sodium Bicarbonate, R8758) and for HL-60 and HMC-1 cells (Iscove’s Modified Dulbecco’s Medium (IMDM) with L-Glutamine, FG0465) were from Sigma-Aldrich (Taufkirchen, Germany), and fetal bovine serum (FCS) Gold (A15-751) from PAA Laboratories (Pasching, Austria). The cell lines HL-60 (promyelocytic leukemia) and U937 (histiocytic lymphoma) were from the European Collection of Authenticated Cell Cultures (ECACC) via Sigma-Aldrich (Taufkirchen, Germany). The HMC-1 cell line (mast cell leukemia) [29] was a gift from Dr. J. H. Butterfield, Mayo Clinic (Rochester, MA, USA).

### 4.2. Isolation of Human Neutrophil Granulocytes

Peripheral venous blood was collected from six healthy donors after informed consent (votum 018-09 of the local ethics committee), and differential blood counts were performed. Neutrophil granulocytes were isolated using the MACSxpress neutrophil isolation kit (Miltenyi Biotec, Bergisch Gladbach, Germany) following the manufacturer’s instructions. Residual erythrocytes were eliminated by hypotonic lysis. Neutrophils were resuspended in PBS with glucose (1 g/L) at a density of 50–100 cells/µL. The purity of the final cell population was analyzed by staining a smear using Pappenheim’s method and counting ≥200 cells using a light microscope. Purity was >99% neutrophils in all isolates.

### 4.3. Cell Culture

U937 and HL-60 cells were propagated in RPMI 1640 and HMC-1 cells in IMDM, each supplemented with 10% FCS, and kept at 37 °C, 5% CO_2_, and 95% humidity; HMC-1 medium additionally contained 4% L-glutamine and 0.02% 1-thioglycerol. Cell density was monitored using a CASY-1 cell counter (Schärfe System, Reutlingen, Germany) and kept at 1–10 × 10^5^ cells/mL.

### 4.4. Cell Lysis

For the quantification and characterization of HNE-like activity in cell lysates, the density of the sample cell suspension was first quantified using a CASY-1 cell counter. Cells were then pelleted in test tubes by centrifugation (400× *g*, 10 min, 4 °C) and washed 3 times with PBS (4 °C). Washed cells were lysed by adding lysis buffer (PBS, 1 M NaCl, 0.1% Tween-20, pH 7.4; ~1000 cells/µL) followed by sonification (4 pulses of 15 s each on ice using a Sonifier B-12; Branson, Danbury, CT, USA) or alternatively by repeated freeze-thawing on dry ice. All lysates were centrifuged (2100× *g*) to remove cell debris before activity measurements were carried out.

### 4.5. Elastase Activity Measurements

The enzymatic activity of HNE was measured using a fluorescence-based assay in 96-well black bottom plates (Corning^®^, 003916, New York, NY, USA). HNE (0.5 nM) or cell lysates (20 µL, ~100 PMN/µL, ~1000 U937/µL, ~5000 HL-60/µL or ~1000 HMC-1/µL) were diluted in assay buffer (200 mM TRIS HCl, 500 mM NaCl, 0.05% Tween-20, pH 8.0). After the addition of the elastase-specific fluorogenic substrate MeOSuc-AAPV-AMC (10 µM), the release of 7-amino-4-methylcoumarin (AMC) was followed over 10 min at 37 °C in a Safire^2^ Multimode Microplate Reader (Tecan, Crailsheim, Germany) at excitation and emission wavelengths of 380 and 460 nm, respectively. AMC (7-Amino-4-methylcoumarin) standard curves were generated by measuring serial dilutions of the fluorophore AMC (1 µM–0 µM) in assay buffer on the same plate and used to calculate nM AMC generation from fluorescence measurements. If necessary, fluorescence data were corrected for AMC bleaching using the decrease of fluorescence over time in samples where HNE activity was completely inhibited.

To determine the amount of HNE in cells, the activity of HNE in lysates was measured in comparison to HNE standards (0.5 nM) on the same plate. The amount of HNE in one million cells was then calculated based on the known cell number of the lysate sample, the molar mass of HNE (29.5 kDa), and the assay volume of 200 µL; the HNE standard of 0.5 nM corresponds to 2.95 ng HNE in the assay volume of 200 µL.

### 4.6. Kinetic Parameters of the Inhibitors

To determine the inhibition constants of reversible inhibitors with isolated HNE and HNE in cell lysates, HNE (0.5 nM) and U937 lysates (1000 cells/µL) were incubated with serial dilutions of the compounds in assay buffer at 37 °C for ≥ 1 h. Subsequently, residual HNE activity was measured as described above and compared to the activity of uninhibited HNE. For compound 5b, a modified assay buffer was used (12 mM HPO_4_^2−^/H_2_PO_4_^−^, 0.637 M NaCl, 2.7 mM KCl, 0.05% Tween 20, pH 7.4) because this inhibitor is unstable in TRIS-HCl. The inhibition curves generated were fitted to Morrisons’s equation to obtain the dissociation equilibrium constant *K*_i_ with pro-Fit (Quantum Soft, Uetikon am See, Switzerland) using a Levenberg–Marquardt algorithm. Similarly, inhibition by irreversible inhibitors was measured after 1 h, and results were fitted using a logistic equation to obtain an IC_50(60min)_.

To determine the association rates (*k*_on_) of the inhibitors, the enzymatic activity of HNE (0.6 nM) was recorded continuously in an SFM25 fluorometer (Biotek Kontron, Neufahrn, Germany) by following the cleavage of MeOSuc-AAPV-AMC (10 µM) at 37 °C. When a constant rate was established (i.e., after 1–5 min), an inhibitor was added, and the reaction followed for up to 60 min. The pseudo-first-order rate constant *k*_obs_ was obtained by fitting the observed presteady-state progress curve to the integrated equation of Morrison [47] with pro-Fit. Apparent second-order rate constants were subsequently calculated as *k*_2_/*K*_i_ = *k*_obs_/I_t_ by linear regression from several independent experiments using a range of concentrations. For reversible inhibitors, the dissociation rate *k*_off_ was subsequentially calculated as *k*_off_ = *K*_i_ × *k*_on_; for irreversible inhibitors, *k*_off_ is ≈ zero. Finally, the halftime of the HNE/inhibitor complex formation and dissociation were calculated as t_1/2,on_ = 0.693/(*k*_ass_ × I_t_ + *k*_off_) and t_1/2,off_ = 0.693/*k*_off_, respectively.

### 4.7. Incubation of Cells with Inhibitors

U937 cells were plated in sterile 96-U-well plates (92697, TPP, Trasadingen, Switzerland) at a density of 100 cells/µL in a cell culture medium (180 µL per well). Increasing concentrations of inhibitors and solvent controls were diluted in a culture medium and added (20 µL/well) to the 96-well plate 26 h, 6 h, 3 h, and 0 h before harvesting and lysing the cells (see below). Plates were kept at 37 °C, 5% CO_2_, and 95% humidity throughout the incubation period. Cell proliferation was checked at harvesting and showed a cell density of an average of 1000 cells/µL. Similarly, freshly isolated PMN were plated at 50–100 cells/µL in PBS (1 g/L glucose) and incubated for 1 h with inhibitors or controls.

For cell lysis, cells were pelleted in the microtiter plates by centrifugation (400× *g*, 10 min, 4 °C) and washed three times in PBS (4 °C). Washed cells were then lysed by adding lysis buffer (PBS, 1 M NaCl, 0.1% Tween-20, pH 7.4) and freeze–thawing on dry ice. Finally, lysates were centrifuged (2100× *g*) to remove cell debris. Enzymatic activity of HNE was determined as described above, and residual activity present after incubation with inhibitors expressed as % of matched solvent controls.

### 4.8. Inhibitor Stability

Inhibitors were prepared as above and incubated for 26 h in RPMI at 37 °C. Residual inhibitor activity was compared to the activity of freshly prepared compounds by measuring the inhibition of HNE.

### 4.9. Cytotoxicity

Cytotoxicity of the inhibitors was determined by analyzing the release of lactate dehydrogenase (LDH) into the cell culture medium using the LDH cytotoxicity kit II (PromoKine, Heidelberg, Germany) or the more sensitive Pierce LDH cytotoxicity assay kit (Thermo Scientific, Darmstadt, Germany) following the manufacturer’s instructions.

### 4.10. Western Blot

Protein immunoblots were performed using 4–20% Tris-glycine gels (Anamed, Groß-Bieberau, Germany), followed by transfer onto a 0.45 µm nitrocellulose membrane (Amersham Protran, GE Healthcare, Frankfurt, Germany) with a Thermo Pierce G2 blotter (Thermo Scientific, Rockford, IL, USA). Membranes were blocked for 2 h with 5% BSA in TBS with 0.05% Tween-20 (TBS-T) and incubated with primary antibody MAB91671 (R&D Systems, Minneapolis, MN, USA) overnight at 4 °C, followed by 5 × washing steps with PBS-T. Thereafter, membranes were incubated with the secondary antibody 115-035-062 (HRP; 1: 20,000) (Jackson ImmunoResearch Europe, Ely, UK) diluted in PBS-T 0.5% skim milk for 1 h at room temperature, followed by 2 × 2 washing steps with PBS-T/PBS. Detection was performed using SuperSignal West Dura Substrate (Thermo Scientific, Darmstadt, Germany) and Amersham Hyperfilm ECL (GE Healthcare, Buckinghamshire, UK), exposing CL-XPosure™ clear-blue X-ray films (Thermo Scientific, Darmstadt, Germany) developed with Carestream GBX processing chemicals (Hartenstein, Würzburg, Germany).

### 4.11. Statistical Analysis

All analyses were performed with pro-fit (version 6.x and 7.x; Quantum Soft, Uetikon am See, Switzerland) or GraphPad Prism (version 8.x–10.x; GraphPad Software Inc., San Diego, CA, USA). Results are presented as mean ± SEM of n ≥ 3 independent experiments if not otherwise stated. Data from experimental groups were compared using analysis of variance and Tukey’s or Dunnett’s multiple comparisons test, where appropriate. A *p*-value of < 0.05 was considered significant.

## Figures and Tables

**Table 1:** Kinetic parameters *K*_i_ and IC_50(60min)_ for the reversible and irreversible inhibitors, respectively, were used in this study to profile isolated HNE and HNE in U937 lysates. Isolated HNE and U937 lysates were incubated with increasing inhibitor concentrations for one hour, and residual HNE activity was measured after the addition of substrate. The inhibition curves were then fitted to Morrisons’s equation and to a logistic equation to obtain *K*_i_ and IC_50(60min)_, respectively. Values are mean ± SD, *n* ≥ 3 individual experiments.

Inhibitor		Isolated HNE		U937 Lysate	
		*K*_i_ [M]	IC_50_ [M]	*K*_i_ [M]	IC_50_ [M]
α_1_-PI	irreversible	-/-	4.6 ± 1.8 × 10^−10^	-/-	5.7 ± 1.5 × 10^−10^
MeOSuc-AAPV-CMK		-/-	1.7 ± 0.3 × 10^−8^	-/-	3.1 ± 0.4 × 10^−8^
Pefabloc SC		-/-	1.3 ± 0.1 × 10^−4^	-/-	1.3 ± 0.1 × 10^−4^
Alvelestat	reversible	9.0 ± 2.0 × 10^−9^	-/-	1.1 ± 0.2 × 10^−8^	-/-
Benzamidine		>1 × 10^−3^	-/-	>1 × 10^−3^	-/-
BPTI		3.9 ± 0.4 × 10^−7^	-/-	3.3 ± 1.4 × 10^−7^	-/-
Cathepsin G Inhibitor		>1 × 10^−5^	-/-	no data	-/-
Compound 5b		2.5 ± 0.8 × 10^−8^	-/-	1.9 ± 0.7 × 10^−8^	-/-
DMP 777		2.5 ± 0.1 × 10^−10^	-/-	3.4 ± 0.7 × 10^−10^	-/-
Eglin C		1.3 ± 0.2 × 10^−11^	-/-	1.9 ± 0.8 × 10^−10^	-/-
GW311616		4.3 ± 0.5 × 10^−10^	-/-	4.9 ± 0.6 × 10^−10^	-/-
Sivelestat		1.7 ± 0.3 × 10^−8^	-/-	1.9 ± 0.4 × 10^−8^	-/-
SSR 69071		3.3 ± 3.5 × 10^−10^	-/-	3.3 ± 2.1 × 10^−10^	-/-

**Table 2:** Kinetics of the inhibition of HNE. The association rate *k*_on_ of the formation of the HNE/inhibitor complex was determined using pre-steady-state kinetics. The dissociation rate *k*_off_ of complex with a reversible inhibitor is derived from the *k*_on_ and the *K*_i_ determined using steady-state kinetics (see Table 1). From these values, the half-time of the complex formation t_1/2_, on at the maximal inhibitor concentration Imax applied to U937 cells, and of its dissociation are calculated. Values are mean ± SD, *n* ≥ 3. * Data from [35], as the reaction with alvelestat is too fast to be recorded precisely with our setup.

Inhibitor		*k*_on_ [M^−1^ s^−1^]	*k*_off_ [s^−1^]	t_1/2, off_ [min]	I_max_ [M]	t_1/2, on_ [s]
α_1_-PI	irreversible	2.1 ± 0.1 × 10^6^	≈0	∞	3 × 10^−6^	1.0 × 10^−1^
MeOSuc-AAPV-CMK		5.5 ± 0.7 × 10^3^	≈0	∞	1 × 10^−4^	1.3
Pefabloc SC		1.0 ± 0.1	≈0	∞	3 × 10^−4^	2.3 × 10^3^
Alvelestat *	reversible	7.0 × 10^6^	5.7 × 10^−2^	0.2	3 × 10^−6^	3.0 × 10^−2^
Compound 5b		1.8 ± 0.2 × 10^5^	4.5 × 10^−3^	3	1 × 10^−5^	3.8 × 10^−1^
DMP 777		4.9 ± 0.4 × 10^5^	1.2 × 10^−4^	96	1 × 10^−6^	1.4
Eglin C		2.9 ± 0.09 × 10^6^	3.8 × 10^−5^	303	1 × 10^−5^	2.0 × 10^−2^
GW311616		7.0 ± 0.2 × 10^4^	3.9 × 10^−5^	295	1 × 10^−6^	9.9
Sivelestat		1.2 ± 0.1 × 10^5^	2.0 × 10^−3^	6	3 × 10^−4^	2.0 × 10^−2^
SSR 69071		2.8 ± 0.06 × 10^5^	9.2 × 10^−5^	125	1.2 × 10^−5^	2.1 × 10^−1^

## Data Availability

All data generated or analyzed during this study are included in this article. Further inquiries can be directed to the corresponding author.

## Supplementary Materials

The following supporting information can be downloaded at https://www.mdpi.com/article/10.3390/ijms25147917/s1.
