# Supplementary material for: Human Neutrophil Elastase: Characterization of Intra- vs. Extracellular Inhibition
Source: Int J Mol Sci. 2024 Jul 19;25(14):7917. doi: 10.3390/ijms25147917 (PMC11276905; doi:10.3390/ijms25147917)
Supplement: Supplementary file 1 [file ijms-25-07917-s001.zip › ijms-3090067-supplementary.pdf]

Supplementary Materials

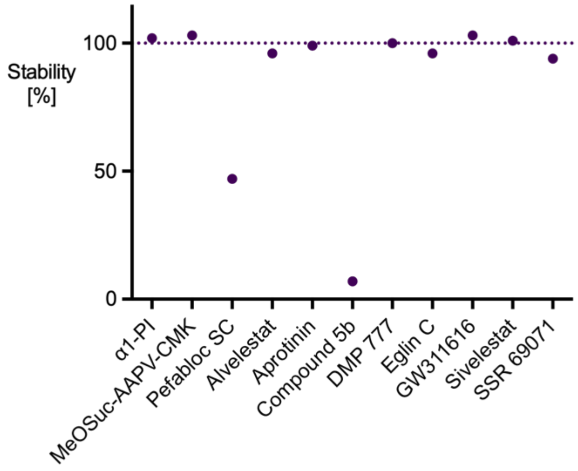

Figure S1: Inhibitor stability. Elastase inhibitors were incubated for 26h in serum-free U937 cell culture medium at 37°. Subsequently, residual compound activity was determined by measuring the inhibition of isolated HNE (0.5 nM) and expressed in % of that of freshly prepared compounds.

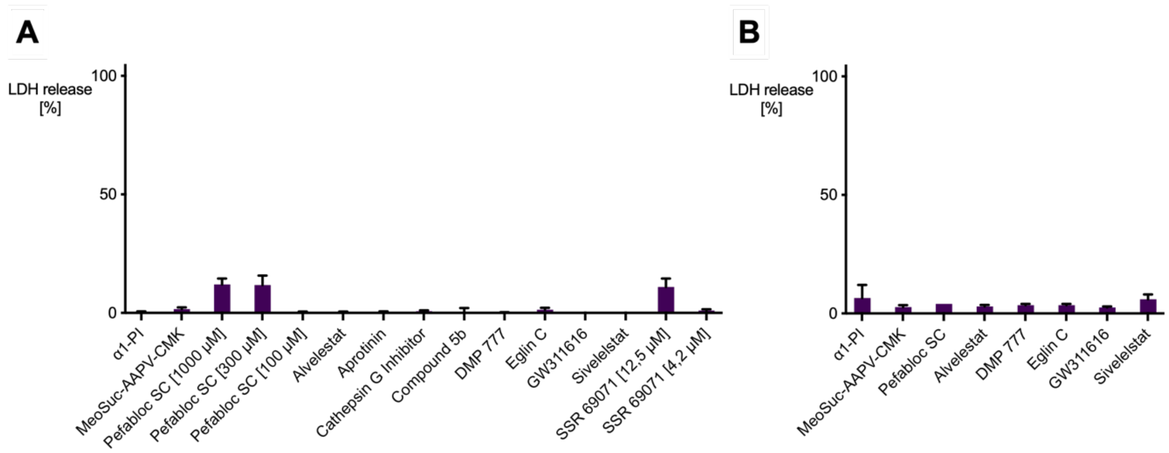

Figure S2: Inhibitor toxicity in U937 cells and PMN. Cytotoxicity of the inhibitors used in Figures 2 and 3 was determined by quantifying the release of LDH into the culture medium after incubation of U937 cells (**A**) and PMN (**B**) for 26h and 1h with the compounds, respectively. If not otherwise stated, only the toxicity of the highest inhibitor concentrations used is shown. Values are presented as % release of total cellular LDH and are mean ± SEM, n = 3.
